# Two Unusual Cases

**Published:** 1873-02-01

**Authors:** Ira Manly

**Affiliations:** Murkezan, Wisconsin


### TWO UNUSUAL CASES.

REPORTED BY IRA MANLY, M.D., MURKEZAN, WISCONSIN.

Case I.—A girl about sixteen years of age, well developed, and fine-looking. As the age of puberty came on she began to be affected with monthly periods of great pelvic distress, but without any menstrual flow. On examination, I found a soft tumor closing up the vagina at the middle portion.

Prof. E. Andrews, of Chicago, being called, punctured the swelling with a trochar, in August, 1865. A large quantity of retained menstrual fluid, of a black color and a tarry consistency, flowed off. The obstruction to the vagina was above the hymen, and thin and membranous. It was incised freely both ways, and a dilating influence kept up for some time by a steel instrument made for the purpose, in the form of a glove-stretcher. In spite of all this, there was formed a firm stricture, whose orifice was not larger than a penholder. Meantime the patient married, and seven years after the operation was delivered of a living child, without special difficulty, the parts dilating spontaneously, so that the stricture was only perceptible by a slight roughness after the delivery.

Case II.—Mrs. ——, aged fifty-four years. In 1863 I amputated a breast for a tumor, the character of which, by previous examination, Dr. Andrews thought would justify the proceeding. Eighteen months since she was attacked with sciatica. The kidneys and bowels performed their functions regularly, and I could find no inter-pelvic cause for the difficulty, and treated on the general plan. She became so well—though I think she was never without some disability in locomotion, and pain—that I did not see her till last December, at which time there was great disability in locomotion, and constant pain inside as well as outside of *both* thighs; general hyperæsthesis of abdomen and lower extremities; bowels constipated, and stomach delicate, and an irregular recurrence of vomiting (this I am inclined to attribute to the use of anodynes). Defication occasions great pain; but I can feel nothing wrong in the bowels or rectum. Notwithstanding the length of time since the breast was removed, I have, of course, looked for a recurrence of the cancer. Three years since, she was complaining that there was a little dullness of the lungs, and indistinctness in the murmur, but it cleared up; they are now entirely well.

The uterus admitted the sound two and a half inches; no misplacement; os healthy; some tenderness on pressure seeming to be connected with the general hyperæsthesis. This soon subsided after wearing a pledget of lint at the os saturated, with bell. ext. and sol. iod. pot.

The patient was stout, and the abdomen pendulous and thick; manipulations external unsatisfactory; could obscurely feel a very small tumor in right inguinal region; suspected hypertrophy, or possibly malignant disease of the ovary—but on the theory that it is related to the diseased breast. The length of time makes me very doubtful of this view of the pathology. Since May, this patient has had anasarcous legs, for which I am not able to account; the bowels are very tympanitic, and so great and long-continued is the pain from any manipulation, that I have not been able to examine sufficiently to say that there is not at present inter-pelvic pressure on the blood-vessels, that causes the effusion.

I have treated the case empirically; hoping it to be functional, have kept her for several days on large doses of bark. Gave largely, for a considerable time, iod. and brom. pot., iron, etc., etc. Farradism aggravated the pain. Have exhausted counter-irritation and endermic remedies, and finally have been driven from every medical appliance, except the hypodermic use of $\frac{1}{2}$ gr. morph. every two hours; and what surprises me is that my patient is worn out and dying, and still I have no definite notion of the pathology of the case.

September 23d.—After writing the above, I learned that my patient, whom I had not seen for some time, was rapidly failing, and I retained it, hoping to get light on the pathology. I had a post-mortem examination on the 10th inst., but have been so much occupied that I could not sooner report the result.

As I approached the body, the extreme shortness of the abdomen made me suppose that the women had failed to remove the clothing, as I had directed; but on examination found the ribs shut down within the illiac crests.

The lungs were entirely healthy, except a few slight adhesions. The heart, thin and fatty, weighed eight ounces; liver completely studded with *cancer*, but tucked up under the edge of the ribs, so that it could not have been felt externally; stomach very thin and translucent; veins very large; cardiac and pyloric orifices perfect; pancreas and spleen healthy; kidneys large, but healthy; intestines the same.

Nothing wrong with uterus; left ovary as large as a bean; right, size of a large hickory-nut.

The spine, from the eighth dorsal vertebræ had an unusual curvature anteriorly, as though it had been forcibly bent, putting the attenuated ligaments on the stretch, so that the rough edges of the bodies could be felt, ready to be forced through. The *processes* were sound, as well as the bodies, so far as superficial dissection could detect. Did not cut down on the cord.

The shape of the spine accounted for the position of the ribs, and shortening of the abdomen.

I cannot say that the distortion of the spine was a pathological condition. She was so fat when I made my inspections, in the early month, that the position of the ribs might have been overlooked.

She had two very bad bed sores, for which I was not entirely to blame, as she steadily refused to lie on an air-cushion; but the pain was no greater after than before this occurrence.

The spinal curve was probably a position instinctively assumed to avoid painful trac_ tion upon some of the ligaments or other tissues in relation with the liver. The hyperæsthesia may have been from the irritation of the great sympathetic nerves, transmitted to the spinal cord by the communicating branches.

